# Social Regulation of Negative Valence Systems During Development

**DOI:** 10.3389/fnsys.2021.828685

**Published:** 2022-01-20

**Authors:** Shannon Hu, Katherine Packard, Maya Opendak

**Affiliations:** Kennedy Krieger Institute, Solomon H. Snyder Department of Neuroscience, The Johns Hopkins University School of Medicine, Baltimore, MD, United States

**Keywords:** stress, attachment, mother-infant dyad, amygdala, learning, social behavior, dopamine

## Abstract

The ability to sense, perceive, and respond appropriately to aversive cues is critical for survival. Conversely, dysfunction in any of these pathway components can lead to heightened avoidance of neutral or rewarding cues, such as social partners. The underlying circuitry mediating both negative valence processing and social behavior is particularly sensitive to early life experience, but mechanisms linking experience to pathology remain elusive. Previous research in humans, rodents, and non-human primates has highlighted the unique neurobiology of the developing infant and the role of the caregiver in mediating the infant’s negative valence circuitry, and the importance of this early social relationship for scaffolding lasting social behavior. In this review, we summarize the current literature on the development of negative valence circuits in the infant and their social regulation by the caregiver following both typical and adversity-rearing. We focus on clinically-relevant research using infant rodents which highlights the amygdala and its interface with the mesolimbic dopamine system through innervation from the ventral tegmental area (VTA) as a locus of dysfunction following early-life adversity. We then describe how these circuits are recruited to perturb life-long social behavior following adversity and propose additional therapeutic targets in these circuits with an eye toward developing age-appropriate interventions.

## Introduction

Social support is associated with positive health outcomes, and research has demonstrated that the presence of a social-support figure can reduce psychological and physiological responses to aversive experiences (Inagaki and Eisenberger, 2012; Hornstein and Eisenberger, 2017). Indeed, the critical need for social support during adverse experiences has been underscored during the COVID-19 pandemic, in which isolated individuals have faced increased adverse mental health outcomes (Sahi et al., 2021). In order for this social support to be effective, the brain-body systems responding to aversive cues, or negative valence circuits, must be functioning appropriately. In a highly dynamic environment, the ability to sense, perceive, and respond appropriately to aversive cues is critical for survival. Conversely, dysfunction in any of these pathway components can lead to impaired threat responses and is a key impairment in disorders such as PTSD and anxiety (Callaghan et al., 2019; Meyer and Lee, 2019; Abend et al., 2020).

Impaired negative valence systems can be damaging not only when an individual fails to avoid a threat, but also if the individual fails to approach potential rewards due to heightened threat responses. As an example, for most species, access to food, protection, and receptive mates requires a motivation to approach social partners, whereas failure to do so prevents access to these key resources. Furthermore, failing to engage in social approach behavior can prevent access to social support during adversity. As noted above, this can lead to increased susceptibility to highly aversive stimuli, chronic stress system engagement, and further maladaptive remodeling of negative valence systems.

The plasticity of these circuits that renders them open to social regulation also renders them vulnerable to perturbation by extreme adverse experiences, such as trauma. Specifically, mounting evidence suggests that these circuits are particularly sensitive to experiences during early development, such as early caregiving adversity (Demers et al., 2018; VanTieghem and Tottenham, 2018; Birnie et al., 2020; Tottenham, 2020). As will be discussed below, species-atypical care can perturb threat responses and undermine the effectiveness of social partners during aversive cues later in life. In this way, the unique impact of social stimuli is two-fold—both scaffolding these developing negative circuits and regulating their function in adulthood. Although these circuits are a critical locus of dysfunction in psychopathology, we have limited understanding of the mechanisms linking early social experience, negative valence circuits, and social behavior. However, recent advances in circuit dissection techniques in infant rodent models have provided much-needed insight into this question.

Here, we review the literature on the development of these circuits, their role in social behavior, and the impact of social experience on their function. We focus on clinically-relevant research using infant rodents which permits circuit dissection techniques to ask, how do these systems develop normally, and how does early trauma impact their lasting function to perturb social behavior? We center our discussion on the early social niche of the altricial infant, the caregiver-infant dyad, as this relationship shows unique impacts on the development of negative valence systems.

### Why Focus on the Infant-Caregiver Relationship?

The role of the social partner in regulating developing negative valence circuits is rooted in the unique relationship between the infant and caregiver across altricial species. In these species, infants are born immature and depend on the caregiver for survival, including the provision of food, shelter, protection, and physiological regulation. John Bowlby’s Attachment Theory drew on observations from comparative research that infants across altricial species work to maintain contact with the attachment figure, with evolution and survival placing heavy selection bias on these infant-caregiver dyads (Bowlby, 1965, 1978). Rapid attachment is a key component of the infant-caregiver relationship early in life. An example is imprinting in birds (Hess, 1962), which demonstrates an example of rapid and robust attachment immediately after birth. Similarly, non-human primates attach to caregivers that provide contact comfort, a process that is required for healthy development (Harlow and Harlow, 1965).

Forming attachments also impacts altricial infants’ interaction with immediate threats in their environment. When faced with threatening cues, the motorically immature infant is unable to fight or flee and instead seeks protection from the caregiver (Coss and Penkunas, 2016). The caregiver also regulates the infant’s neurobehavioral responses to threats—a process termed social buffering, whereby the caregiver acts as a “safe haven” for the young and attenuates the child’s fear response to potentially threatening or stressful stimuli (Hennessy et al., 2009; Gunnar et al., 2015; Doom et al., 2017; Hornstein and Eisenberger, 2017). Social buffering has been observed across species, including rodents, non-human primates, and humans.

## Neural Circuits Supporting Infant Social Buffering

The neural circuits supporting social buffering involve suppression of cortisol, or its non-human animal analog corticosterone (CORT), from the hypo-thalamic-pituitary axis during stress (Hennessy et al., 2006; Faustino et al., 2017; Howell et al., 2017). This attenuation in turn impacts multiple brain regions typically engaged during threat processing, particularly the amygdala (Fuzzo et al., 2015; Opendak et al., 2019; Robinson-Drummer et al., 2019). The activation of the amygdala is greatly attenuated in human children during a mildly stressful task when the caregiver is present (Gunnar and Donzella, 2002; Gee et al., 2014). Additionally, the mechanisms underlying the recruitment of the amygdala in threat acquisition, consolidation, and expression are believed to be well-conserved across mammalian species (Maren and Fanselow, 1996; Fanselow and LeDoux, 1999; Phelps and LeDoux, 2005; Johansen et al., 2011).

Rodent research has helped elucidate how and when this brain region becomes involved in threat processing, and the impact of caregivers on this process. In typically developing infant rats, the amygdala is not functionally engaged in threat learning during the first few days of life; manipulations to inhibit activity in the amygdala have no effect prior to postnatal (PN) day 10 (Raineki et al., 2009). As a result, these infant rats, or pups, do not form conditioned aversions to stimuli with negative valence, though they are capable of experiencing physical pain and forming amygdala-independent aversions (Rudy and Cheatle, 1983; Blass, 1997; Barr, 2011). This early amygdala inactivity, combined with the unique functioning of the infant odor processing circuitry (anterior piriform cortex, olfactory bulb, and locus coeruleus), means that pups learn to approach any cue associated with the caregiver, even if that cue is aversive (Sullivan et al., 1992, 2000; Sullivan and Wilson, 1994).

Gradual engagement of the amygdala in threat processing occurs alongside developmental changes in endogenous CORT levels (Thompson et al., 2008). In early life, plasma CORT levels are typically very low in rats, a phenomenon termed the stress hyporesponsive period (SHRP; Levine et al., 1985, 1988; Levine, 2001; Hennessy et al., 2006). The gradual rise in CORT as the pup ages has been found to be causal in inducing plasticity in the amygdala and conferring the ability to learn threat: exogenous systemic injection of CORT at PN8 prematurely recruits the amygdala in threat learning processes while adrenalectomy in PN12 rats prevents threat expression and amygdala activation (Upton and Sullivan, 2010). The molecular mechanism of this plasticity has also been traced to the trafficking of GluA1 and GluA2 AMPA receptor subunits to synapses in the basolateral amygdala (BLA) and activation of upstream protein kinases protein kinase M zeta (PKMζ) and iota/lambda (PKCι/λ) in response to elevated CORT. The increased GluA1/2 ratio stabilizes synapses in the amygdala and is required for the formation of threat memories (Huganir and Nicoll, 2013; Oliver et al., 2016; Opendak et al., 2018).

Endogenous CORT levels increase around PN10, an age at which rat pups begin to wander away from the nest and nibble solid food (Sullivan and Holman, 2010; Upton and Sullivan, 2010). This change is accompanied by a transition from the sensitive period for attachment formation to a period when amygdala-dependent threat learning can occur to promote avoidant responses to threat-predicting cues. A similar transition occurs in humans when fear of strangers emerges in young children (Callaghan et al., 2019). One key feature of this developmental window is the new ability of the caregiver to regulate the pup’s stress response, whereby the presence of the caregiver or caregiver odor robustly decreases amygdala activity and plasma corticosterone levels, and actively promotes attachment behavior (Moriceau and Sullivan, 2006). The caregiver’s presence switches what is learned about the stimulus—specifically, caregiver presence during conditioning switches learned aversions to learned preferences during this period, a striking reversal which has recently been documented in children (Tottenham et al., 2019). From PN16 onwards in rats, the power of the caregiver or social partner transitions to a more modulatory function, similar to the effect of social partners in adulthood (Eisenberger et al., 2011; Hornstein and Eisenberger, 2017). A similar shift has been observed in human children, where the caregiver has the power to regulate amygdala-PFC connectivity in younger children but not adolescents (Gee et al., 2014).

Caregiver regulation of infant threat processing affects the midbrain dopamine (DA) circuit involving the amygdala. Decades of microdialysis studies have shown that caregiver inputs such as presence and milk decrease DA release from the ventral tegmental area (VTA), the primary source of dopamine to the amygdala (Tamborski et al., 1990; Andersen et al., 1992; Kehoe et al., 1998). Furthermore, caregiver presence prevents DA release in the BLA during threat (Barr et al., 2009), while DA has been shown to be necessary for BLA long-term plasticity in adults to promote learning (Rosenkranz and Grace, 2002; Lorétan et al., 2004; Kienast et al., 2008; Fadok et al., 2009; Tye et al., 2010; Ng et al., 2018). During the period when the caregiver can toggle preference vs. aversion in rat pups (PN10-15), the caregiver has been shown to regulate the broader mesolimbic dopamine circuit, including the VTA, BLA, and nucleus accumbens (Opendak et al., 2019). As pups develop past weaning (>PN21), the BLA circuit partners impacted by the mother switches from the VTA to the prefrontal cortex (Robinson-Drummer et al., 2019). In post-weaning pups, maternal presence buffers mPFC reactivity to threats, similar to the effect observed in young adults viewing photos of attachment figures during exposure to stressors (Eisenberger et al., 2011). Interestingly, the lateral habenula (LHb), a brain region that shows functional engagement during aversive cues and inhibits DA release from the VTA, continues to be impacted by caregiver presence even in older PN28 pups (Packard et al., 2021).

Taken together, these data suggest that infant negative valence systems have a unique function. Instead of processing threatening or aversive cues to generate avoidance responses, these circuits interpret the caregiver as the ultimate arbiter of ambiguous cues. As a result, the caregiver has immense control over the infant’s emotional environment at an age when these brain circuits are still developing.

## Early Life Adversity Degrades Social Regulation of Negative Valence Circuits

As noted above, the adaptive features of approaching the caregiver ensure that attachments are formed, regardless of the quality of the care received. However, abusive and/or neglectful care can impact the quality of attachment that is formed and in turn, the ability of the caregiver to regulate the infant. For example, caregivers in stressful or neglectful rearing conditions have impaired ability to decrease infant stress responses in the Strange Situation Procedure, a canonical laboratory procedure measuring attachment quality in children (Ainsworth and Bell, 1970; Nachmias et al., 1996). The quality of attachment can also be evaluated by the robustness of the child’s oscillatory responses to caregiver cues, as measured with EEG (Stamoulis et al., 2015; Perone and Gartstein, 2019; Pratt et al., 2019). Children reared in adverse caregiving conditions also show decreased fMRI response in the amygdala and prefrontal cortex to caregiver cues (Callaghan et al., 2019; Tottenham, 2020).

Recent work in rodents has dissected specific features of compromised caregiver buffering of infant negative valence systems. When pups were reared using the Scarcity-Adversity paradigm from PN8-12, in which mother rats are given limited bedding and roughly handle pups, we observed degraded neurobehavioral impact of reunion with a caregiver at PN13 following separation in a rodent Strange Situation procedure—similar to effects observed in children. However, we also were able to record cortical oscillations during adversity-rearing itself and observed that within this context, nurturing maternal inputs, such as grooming and milk ejection, failed to produce expected changes in cortical oscillations (Opendak et al., 2020). These results suggest that impaired processing of nurturing cues, rather than adverse care itself, may drive degraded attachment.

Work in rodent pups has also permitted deconstruction of naturalistic adversity conditions to further identify mechanisms linking experience to the outcome. For instance, the mere presence of a caregiver during repeated adversity was sufficient to produce neurobehavioral deficits in pups, including amygdala dysfunction and social behavior impairments observed at PN13. Specifically, these impairments were observed if pups experienced repeated shock or exogenous CORT administration in the presence of an anesthetized mother, but not alone, from PN8-12 (Raineki et al., 2019; Opendak et al., 2021).

We now understand that impaired social buffering involves dopamine release in the amygdala from the VTA. This mesolimbic dopamine—BLA interface appears to integrate the effects of development, experience, and social buffering (Atzil et al., 2017; Opendak et al., 2019). In typically developing pups at PN12-14, caregiver presence during threat buffers VTA, which is disrupted by adversity-rearing (Opendak et al., 2019). Furthermore, microdialysis in pups shows that repeated shock in the presence of the caregiver increases DA in BLA, and results in failure of the caregiver to buffer amygdala plasticity in response to threat (Opendak et al., 2021). Collectively, these findings define the necessary combination of stress and caregiver presence to disrupt caregiver regulation of negative valence circuits and highlight that adverse maternal behavior *per se* is not necessary to recapitulate the neurobehavioral effects of adversity-rearing.

## Implications for Social Behavior

Early social regulation of these negative valence circuits, such as the mesolimbic DA-BLA interface, scaffolds function of these circuits in social behavior across the lifespan. Cross-species data suggest that the amygdala function in social behavior shows developmental transitions. In humans, the amygdala is responsive to fearful stimuli as early as 6–9 months of age (Jessen and Grossmann, 2014; Graham et al., 2016), but its role in social behavior is less well understood. In non-human primates, bilateral amygdala lesions impair social behavior in adulthood but not infancy (Bachevalier et al., 2001; Goursaud and Bachevalier, 2007; Raper et al., 2014). Similarly, pharmacological and optogenetic lesion studies in rodents suggest that amygdala engagement is atypical in infant social behavior (Raineki et al., 2019; Opendak et al., 2021). This early lack of amygdala involvement is in line with the unique ecological niche of the infant which promotes an early social behavior bias toward approaching the mother under threat and safety (Fox et al., 2005; Moriceau and Sullivan, 2006; Coss and Penkunas, 2016; Shultz et al., 2018).

**Figure 1:**
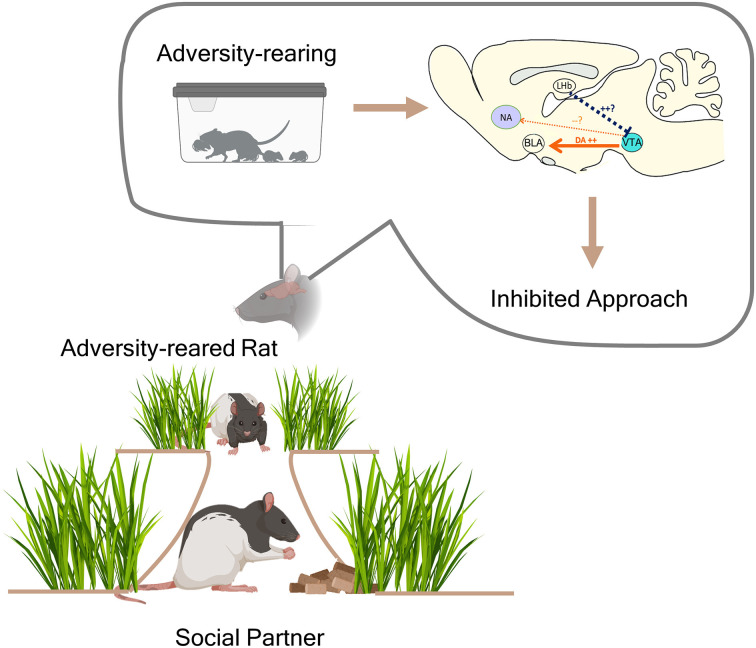
Remodeled negative valence circuits inhibit social behavior after adversity. Following early life adversity with a caregiver, increased dopaminergic innervation from the ventral tegmental area (VTA) to the basolateral amygdala (BLA) results in decreased social approach toward a novel peer. Measurements during the adversity itself show that whereas caregiver presence typically suppresses dopamine (DA) release in the BLA during threat exposure, repeated adversity with the caregiver is accompanied by enhanced amygdala reactivity and DA release in the caregiver’s presence. This suggests that negative valence circuits are engaged in processing the caregiver and this produces lasting hyperactivity of these circuits in responding to social partners. Research now turns to the lateral habenula (LHb) as the site of dysfunction in social behavior, as this region inhibits DA release from the VTA to the classical reward locus of the nucleus accumbens. Based on recent work, we propose a model whereby early adversity produces LHb hyperactivity (blue dashed line) and inhibited DA release to the accumbens (NA); combined with enhanced DA release in the VTA, these circuit changes decrease the rewarding salience of social partners.

Recent circuit dissection work in rodent pups has highlighted the role of dopaminergic projections from the VTA to the BLA in modulating developmentally-appropriate social behavior (see Figure 1; Opendak et al., 2021). Optogenetic inhibition of the BLA and VTA terminals in the BLA increased social approach in older (PN23) but not younger pups (PN14), suggesting these regions are not involved in inhibiting social behavior in young pups. This work also showed that early life adversity can prematurely engage the amygdala to put a brake on the social approach towards the caregiver. More specifically, dopamine release in the BLA is both necessary and sufficient in producing social avoidance behaviors following adversity. Early-life adversity with the mother (both naturalistic Scarcity-Adversity and repeated shock with mom) from PN8-12 increased dopamine in the pup BLA, and its blockade through pharmacological and optogenetic techniques rescued typical social behavior at pre and post-weaning ages. Conversely, optogenetic stimulation of the VTA to BLA circuit in control-reared pups inhibited typical social approach at PN14. Taken together, these data show that dopaminergic innervation of the BLA is atypical during early social behavior and recruitment of this circuit transitions a system biasing social approach toward the caregiver toward one favoring a balance of approach and avoidance as infants mature.

## Remodeling of Extended Negative Valence Circuits

The VTA-BLA interface does not exist in isolation, and many mesolimbic structures interface with the VTA to promote adaptive social behavior. Research now turns to circuit partners of the VTA and their specific roles in development. One such circuit partner is the lateral habenula, a small, phylogenetically conserved structure in the posterior-dorsal-medial end of the thalamus with rich connections to both prefrontal cortex and midbrain monoaminergic systems (Hu et al., 2020). As mentioned above, this brain region, considered an “anti-reward” center, responds to aversive cues and sends projections to inhibit DA release from the VTA (Proulx et al., 2014; Baker et al., 2015). Hyperactivity in this structure has been consistently implicated in the pathophysiology of depression, suggesting it is likely a target of early trauma (Simmons et al., 2020). Our recent research on this circuit has found that the LHb is engaged in threat processing at a pre-weaning age (PN18) in both typically and adversity-reared pups. However, previous adversity-rearing reduced caregiver buffering of the LHb during threat presentation in post-weaning animals (Packard et al., 2021). Early life adversity also significantly alters functional connectivity patterns between the LHb and downstream circuit partners such as the VTA and substantia nigra, producing a more adult-like connectivity profile. The dysregulated LHb connectivity and LHb hyperactivity of rats experiencing early life stress suggest there may be greater inhibition of downstream targets, such as the VTA, and heightened aversion to potentially threatening stimuli. However, further dissection of specific projections and cell types mediating these differences is needed.

## Could Some Changes to Social Behavior Be Adaptive?

While many studies are quick to highlight perceived deficits in children experiencing early life adversity, it should be noted that many changes in functioning may reflect an optimized behavioral strategy (Ellis et al., 2017, 2020). Indeed, taking a more avoidant approach following repeated exposure to an aversive social environment may be adaptive in some circumstances. Such optimizations, called “hidden talents,” may require specialized testing to uncover and are a fundamental framework to prevent further stigmatization of children raised in adverse environments. Additional examples include individual differences in measures of resilience (Masten et al., 2004; Southwick et al., 2014) and observations of post-traumatic growth (Tedeschi and Calhoun, 2004) following early life adversity across species. This concept of circuit optimization to suit environmental demands has been proposed not only for social behavior circuits but also for others involved in threat processing and reward-seeking (Opendak et al., 2017; Cushman et al., 2021; Hanson et al., 2021).

## Concluding Remarks

Disrupted social behavior is a core symptom of many psychiatric and developmental disorders, including autism, anxiety, and depression (Crawley, 2004; Miller, 2007; Kennedy and Adolphs, 2012; Patel et al., 2019). The ontogeny of these disorders is poorly understood, but clinical research has identified early adversity as a significant risk factor in the scaffolding of lifelong social deficits (Teicher et al., 2002, 2003; Raineki et al., 2012, 2015; Tottenham, 2012; Gee et al., 2013; Malter Cohen et al., 2013; Hanson et al., 2015). More recent evidence suggests that adversity within the social context of the attachment figure renders the infant uniquely vulnerable (Raineki et al., 2019; Opendak et al., 2021). Using invasive circuit dissection techniques previously impossible in pups, here we review how the maternal signal becomes compromised during repeated social adversity and highlight how this may impact social behavior through perturbation of negative valence circuits. This work has highlighted the mesolimbic dopamine interface with the amygdala as a site dysfunction in both caregiver regulation of the infant and infant social behavior. Future work targeting this circuit and upstream regions, such as the habenula, will be critical for developing age-appropriate interventions following early-life adversity.

## Author Contributions

SH, KP, and MO wrote the review. All authors contributed to the article and approved the submitted version.

## Funding

We would like to thank National Institutes of Health (NIH) for funding of this research (BRAIN R00MH124434) and the Brain and Behavior Research Foundation (Young Investigator Grant to MO).

## Conflict of Interest

The authors declare that the research was conducted in the absence of any commercial or financial relationships that could be construed as a potential conflict of interest.

## Publisher’s Note

All claims expressed in this article are solely those of the authors and do not necessarily represent those of their affiliated organizations, or those of the publisher, the editors and the reviewers. Any product that may be evaluated in this article, or claim that may be made by its manufacturer, is not guaranteed or endorsed by the publisher.
